# Profile and outcomes of Parkinson's disease deep brain stimulation in a public tertiary center in Brazil: retrospective cohort

**DOI:** 10.1055/s-0046-1825523

**Published:** 2026-07-21

**Authors:** Juan Sebastián Sánchez-León, Alexandre Baldissera, Giovana Zazo Guidio, Ingrid Lorena da Silva Gomes, Eduardo Drews Amorim, Leonardo Bedatti Koehler, Thomas Hugentobler Schlickmann, Thais Lampert Monte, Gabriela Magalhães Pereira, Paulo Petry Oppitz, Eduardo Goellner, Carlos Roberto de Mello Rieder, Artur Francisco Schumacher Schuh, Bruno Samuel Fraiman de Oliveira, Daniel Teixeira dos Santos

**Affiliations:** 1 Hospital de Clínicas de Porto Alegre, Departamento de Neurologia, Porto Alegre RS, Brazil.; 2 Universidade Federal do Rio Grande do Sul, Porto Alegre RS, Brazil.; 3 Hospital de Clínicas de Porto Alegre, Research Organization Registry (ROR), Porto Alegre RS, Brazil.; 4 Hospital de Clínicas de Porto Alegre, Departamento de Neurocirurgia, Porto Alegre RS, Brazil.; 5 Hospital Santa Casa de Porto Alegre, Departamento de Neurologia, Porto Alegre RS, Brazil.

**Keywords:** Parkinson Disease, Deep Brain Stimulation, Health Systems

## Abstract

**Background:**

Deep brain stimulation (DBS) is an established surgical therapy for advanced Parkinson's disease (PD), but, in Latin America, access remains restricted, and local data are limited.

**Objective:**

To describe the demographic, clinical, and procedural profile, as well as the treatment outcomes and perioperative complications, of patients undergoing DBS at a Brazilian reference center.

**Methods:**

We conducted a retrospective observational study including 101 patients with PD who underwent bilateral DBS implantation between 2012 and 2024 at Hospital de Clínicas de Porto Alegre, Brazil, within the public healthcare system.

**Results:**

The mean age at surgery was of 62.2 years; 63.4% of the patients were male. The mean ages at symptom onset, diagnosis, DBS indication, and surgery were of 43.1, 45.7, 54.5, and 56.7 years respectively. On average, the patients presented 10.9 years of disease duration when surgery was performed. Motor fluctuations (53.5%) and dyskinesias (41.6%) were the most common indications. The subthalamic nucleus (STN) was the predominant target (97%). Mild complications included wound infection (7.9%), while severe complications, such as lead migration, intracranial hemorrhage, or deep infection occurred in 6.9%. Levodopa equivalent daily dose (LEDD) was the primary outcome used to assess postoperative change; it was significantly reduced by ∼ 25% after DBS, from 1,885.6 to 1,406.7 mg at 12 months (Wilcoxon *p* < 0.001).

**Conclusion:**

In the cohort of the present study, DBS was safe and enabled a sustained reduction in dopaminergic medication during follow-up. Despite these benefits, delays in surgery, limited access through the public health system, and device-availability issues continue to limit broader implementation, highlighting persistent structural inequalities in Latin America.

## INTRODUCTION

Parkinson's disease (PD) is a leading neurodegenerative disorder characterized by progressive motor and non-motor symptoms which significantly impact quality of life. 1 Although often associated with aging, the global burden of PD is increasing; in Brazil, current estimates suggest that approximately 535,999 individuals were affected in 2024, with projections indicating this number may surge to 1,250,638 by 2060, with a higher prevalence observed among men and no significant differences in prevalence across Brazilian states. 2 In addition, the estimated mean annual cost per person with PD was of US$ 4,020.48, predominantly driven by direct healthcare costs (63%) and indirect costs (36%), with a substantial proportion of expenses (49%) borne directly by patients and their families. 3 Notably, patients have also reported greater mobility impairment, including the need for walking assistance. Despite these indicators of disease burden, PD may remain underdiagnosed due to multiple contributing factors. 2 These figures underscore the urgent need for region–specific research to guide clinical management, resource allocation, and health policy planning for PD care in Latin America.

Deep brain stimulation (DBS) is a well-established surgical treatment for advanced PD, particularly in patients with motor fluctuations, dyskinesias, or medication-refractory tremor. 4 Despite its proven efficacy, access to DBS remains limited in many low- and middle-income countries due to logistical, financial, and systemic barriers. 5 6 In Brazil's Unified Health System (Sistema Único de Saúde, SUS, in Portuguese), DBS is offered primarily in high-complexity referral centers, often resulting in long waiting lists and delayed surgical intervention.

Understanding the epidemiological profile of patients who undergo DBS, as well as other key variables, such as the time until diagnosis, time until surgery, surgical indication, stimulation programming patterns, and perioperative complications, is essential to generate data that reflect our local reality. These findings can serve as a foundation for the development of context-appropriate protocols and help to increase the visibility of this therapeutic option, which still faces significant access limitations across Latin America.

## METHODS

### Study design and patients

We conducted a retrospective, observational, descriptive study based on a preexisting institutional database, including 101 patients with PD who underwent DBS at a reference center in Brazil: Hospital de Clínicas de Porto Alegre (HCPA), which is accredited to perform DBS procedures within the SUS, with funding from the government of the state of Rio Grande do Sul. All patients with PD fulfilled the UK Parkinson's Disease Society Brain Bank Criteria. 7 The current study was reviewed and approved by the HCPA Ethics Committee (Certificate of Presentation of Ethical Appreciation number: 2024-0130). The Strengthening the Reporting of Observational Studies in Epidemiology (STROBE) statement was followed in the reporting of the present study. 8

The study included patients with a diagnosis of PD who underwent DBS implantation since 2012, when the surgery started being offered through the SUS. Clinical data were collected from patient's electronic medical records available in the Aplicativos para Gestão dos Hospitais Universitários (AGHUse; open source) system (used in HCPA). Patients included in the study were those who underwent DBS until the end of 2024, with complete records in the system and a minimum follow-up of 3 months after the procedure. The decision to use a 3-month minimum postoperative interval was made to optimize the sample size. We included 101 of the 126 patients who underwent DBS implantation at our center. Patients with incomplete records (n = 12) and insufficient follow-up period (n = 13) were excluded from the analysis; several of these individuals continued their postoperative care with their primary neurologist at external centers.

The variables analyzed included demographic and clinical characteristics: age at surgery, sex, origin (Porto Alegre versus other municipalities), ethnicity, family history of PD, comorbidities, and results of genetic investigations (investigated and uninvestigated patients, and among the investigated, those with a positive result associated with a pathogenic or likely pathogenic variant for PD). We also collected variables related to the disease, including age at: symptom onset, diagnosis, and DBS indication and implantation. Finally, procedure-related variables were recorded, such as indication for DBS: motor fluctuations (non-dyskinetic and dyskinesias), refractory tremor, and levodopa intolerance; perioperative complications: mild and severe complications; type of battery: rechargeable or non-rechargeable; stimulation target: STN or globus pallidus internus (GPi); DBS programming: monopolar (conventional programming) and other programming strategies (including alternative conventional configurations, such as bipolar and double monopolar, and advanced programming using interleaving stimulation); 9 and battery-depletion events: a serious complication in a scenario associated with limited availability of replacement devices for patients with non-rechargeable systems.

Additionally, the levodopa equivalent daily dose (LEDD) was compared before and after surgery, and it was calculated by adding the levodopa dose and the doses of other antiparkinsonian medications, each multiplied by its conversion factor according to the recommendations of the Movement Disorders Society (MDS). The score on the motor scale of the Unified Parkinson's Disease Rating Scale (UPDRS) and quality of life were not used for comparison due to lack of data. 10

The continuous variables were expressed as mean and standard deviation values, while the categorical variables (comorbidities, surgical indication, and perioperative complications) were expressed as frequencies and percentages. No multivariate analyses were planned due to the retrospective design and incomplete data.

### Statistical analyses

The data were organized in a Microsoft Excel (Microsoft Corp.) spreadsheet and subsequently exported for analysis. Statistical processing and graphical representations were performed using Python (free and open source) software, version 3.6.9, within the Google Colab (Alphabet Inc.) environment.

The analyses were primarily descriptive, aimed at characterizing the demographic, clinical, and procedural features of the cohort. For the comparison of LEDD before surgery and at 12 months after DBS, the Wilcoxon signed-rank test was applied. Two-tailed *p* -values < 0.05 were considered statistically significant.

## RESULTS

### Descriptive statistics

**Figure 1:**
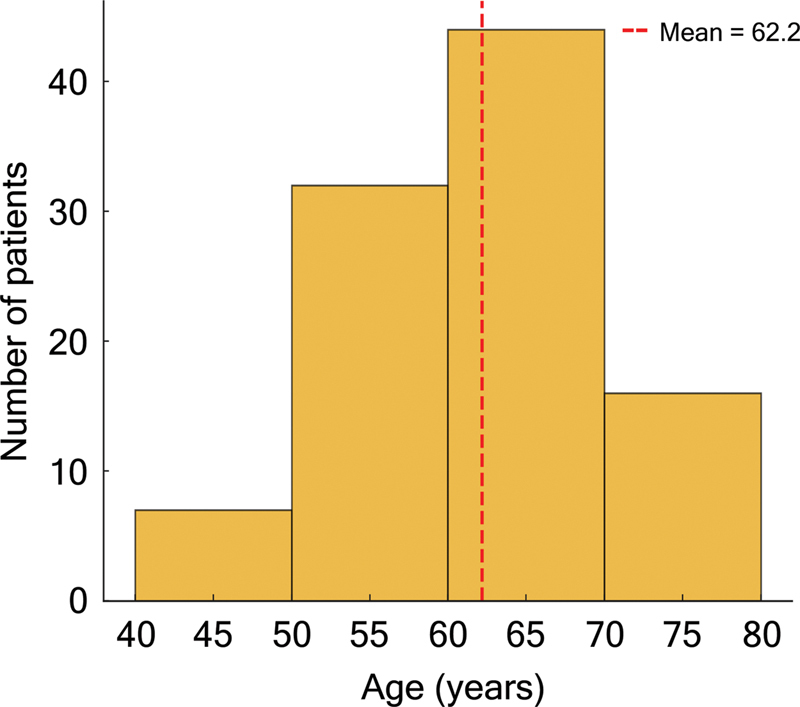
Histogram of the age distribution of patients undergoing deep brain stimulation (n = 101).

Sociodemographic and clinical data are presented in ( Table 1 ). We included 101 patients (out of 126 submitted to surgery) with a mean age of 62.17 years ( Figure 1 ); 63.36% of them were male, and 97.03% were white. The main city of residence was Porto Alegre, the state capital, with 20.79% of patients living there. A total of 93.07% of the patients reported no family history of PD. The most frequent comorbidities were psychiatric disorders, including depression (53.46%) and anxiety (41.58%). In addition, 31.68% of the patients had hypertension. Regarding genetic testing, 36.63% of the patients had undergone it, 5.94% of whom were found to carry variants associated with PD ( Table 1 ). Data collection was conducted in 2024, when 81.19% of the patients were alive.

**Table 1:** Sociodemographic and clinical data (n = 101)

Variables		
Mean age (years)	62.17 ± 8.57	
Male gender: n (%)	64	63.36
Currently residing in Porto Alegre: n (%)	21	20.79
White ethnicity: n (%)	98	97.03
Mean schooling (years)	8.7 ± 2.9	
Negative family history of Parkinson's disease: n (%)	94	93.07
Patients with comorbidities: n (%)	70	69.39
Depression: n (%)	54	53.46
Anxiety: n (%)	42	41.58
High blood pressure: n (%)	32	31.68
Cognitive disorder: n (%)	16	15.84
Smoking: n (%)	11	10.89
Diabetes mellitus: n (%)	7	6.93
Dyslipidemia: n (%)	6	5.94
Alcoholism: n (%)	3	2.97
*Genetic status*		
Genetic status not investigated: n (%)	64	63.37
Patients with detected pathogenic variant: n (%)*	6	5.94
Patient's currently alive	82	81.19

Note: *Patient variants: *LRRK2* : 2; *GBA* : 2; *PARK2* : 1; *CSF1R* : 1; *VPS13C* : 1; and *PRKN* : 1.

### Timeline of PD and DBS surgery

**Figure 2:**
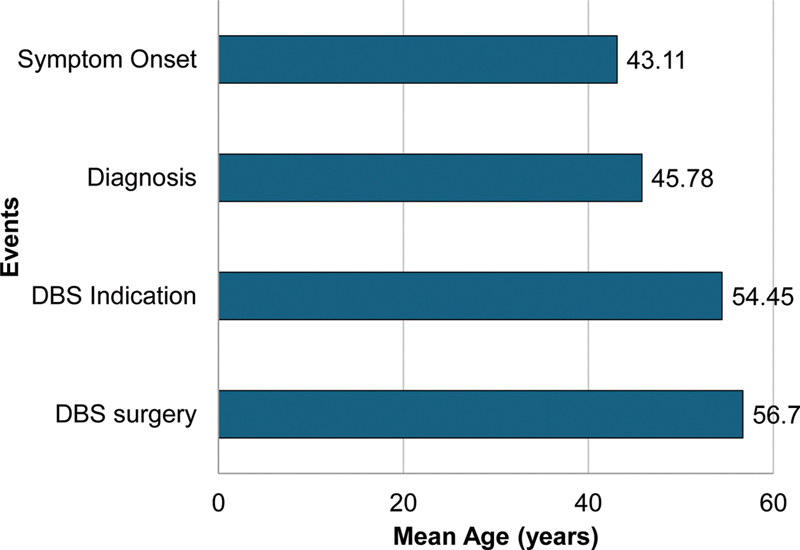
Timeline of Parkinson's disease and deep brain stimulation.

Aspects pertaining to the timeline are presented in ( Figure 2 ). The mean age at symptom onset was of 43.11 years, and, at diagnosis, it was of 45.7 years. The mean age at DBS indication was 5 of 4.45 years, with the surgery performed at a mean age of 56.70 years. On average, there was a 10.92-year time window from diagnosis to DBS placement and a 2.25-year delay from the time of indication to the performance of the surgery.

### DBS procedure and outcomes

Data regarding the DBS procedure and outcomes are presented in ( Table 2 ). We performed an average of 0.87 surgeries per month (126 surgeries throughout 144 months). A total of 57.40% of the patients had more than 1 indication for DBS. Non-dyskinetic motor fluctuations were the most prevalent indication (53.46%), followed by dyskinesias (41.58%). Only 2.97% of the patients underwent DBS targeting the GPi.

**Table 2:** Deep brain stimulation procedure and outcomes (n = 101)

Variables: n (%)			
D **eep brain stimulation** indication	Documented indication	98	97.03
	Two or more indications	58	57.40
	Motor fluctuations	78	53.46
	Dyskinesias	61	41.58
	Refractory tremor	23	31.68
	Intolerance to medications	12	15.84
*Surgical target: subthalamic nucleus*		98	97.03%
Perioperative complications	Total perioperative complications	15	14.85
	Major complications*	7	6.93
	Minor complication: skin infection	8	7.92
*Battery type, rechargeable*		61	60.40%
Battery depletion events	Total battery depletion events	10	9.90
	> 1 battery depletion event	5	4.95
	Event requiring hospitalization	4	3.96
Deep brain stimulation programming: monopolar		63	62.38

Note: *Lead migration: 3 patients; intracranial hemorrhage: 2 patients; and deep lead infection and/or meningitis: 2 patients.

The most frequent minor perioperative complication was skin infection (7.92%). Major complications occurred in 6.93% of the patients: 3 with lead migration, 2 with intracranial hemorrhage, and 2 with cerebritis and/or meningitis. Given the context of resource limitations, not all patients that need to replace their batteries receive them in a timely manner, which is the reason why 9.90% of the patients experienced total battery depletion, 4.95% of whom needed more than 1 battery replacement; in 3.96% of the cases, hospitalization was required: 3 due to DBS withdrawal syndrome, and the other due to a skin lesion that led to generator exposure and subsequent device damage.

At the time of data collection, 62.38% of the patients remained on monopolar DBS programming, with a mean DBS duration of 4.7 years. Patients using advanced DBS programming had a mean duration of 6.3 years. Ultimately, of the 89.1% of the 101 patients had available paired preoperative and 12-month postoperative LEDD data. The mean preoperative LEDD was of 1,885.09 ± 852.48 mg, and the mean postoperative LEDD was of 1,406.71 ± 625.61 mg, corresponding to an approximate 25% reduction. The median LEDD difference was of -300 (interquartile range [IQR]: 0 − 875) mg, with a mean reduction of 478.38 ± 660.37 mg. This change was statistically significant (Wilcoxon signed-rank test; *p* < 0.001). However, pre- and postoperative comparisons of motor outcomes and quality-of-life measures were not systematically available, which limits a comprehensive interpretation of the impact of the intervention.

## DISCUSSION

The current study aimed to describe the experience of a reference center for DBS surgery in the context of a resource-limited setting of the SUS. Between 2012 and 2024, 126 surgeries were performed, averaging 0.87 a month, a number that reflects the limited availability of operating room slots and the restricted number of specialized professionals capable of performing the procedure. The mean disease duration for those undergoing DBS was of 10.92 years, and it took an average of 2.25 years between the procedure being indicated and being effectively performed. The most common indications were motor fluctuations (dyskinetic and non-dyskinetic), and the STN was the most common surgical target. We observed a 25% reduction in total LEDD over a period of 12 months, and the incidence of serious surgical complications remained within international safety benchmarks. Of note, 10/101 patients (9.9%) experienced battery depletion due to delays in obtaining replacement devices. The current is one of the first studies to describe real-world experience and outcomes of DBS in a resource-limited setting in Brazil and Latin America. Regarding the global context, we have reviewed several patient series from the United States, Europe, and Asia; 11 12 13 14 these studies are characterized by significantly larger case volumes, with analyses predominantly focusing on motor improvement and quality of life assessed via pre- and postoeprative scales. Consequently, making a strict comparison with the present study is challenging.

Only limited data are available regarding the reality of DBS surgery in the Latin American population. The high cost of the procedure, combined with the limited availability of specialized teams to manage these patients, has contributed to the scarcity of information and research in our region. 6 Consequently, most of the existing evidence originates from the United States, Europe, and Asia, where the highest-volume DBS centers are located. 15 In the present study, we found a rate of 0.87 surgeries per month, lower than that reported in a global study 16 evaluating DBS variability, in which the average number of surgeries performed per month was of 3.3 (range: 0–15), suggesting that this difference in surgical volume may be associated with differences in resources and accessibility, as previously mentioned, and no comparable data are available from centers in Latin America. Notably, the mean duration of institutional experience in that global study 16 was of 11 years, comparable to that of our center, which at the time of data collection had 12 years of experience with DBS.

In our cohort, surgeries were performed at a mean of 10.92 years after diagnosis, which is consistent with previous studies 13 17 from the United Kingdom and the United States reporting that DBS is typically performed 10–15 years after diagnosis, However, current evidence supports earlier intervention, demonstrating a benefit of surgery in patients with approximately 7 years of PD duration, 14 18 as illustrated by a Chinese multicenter study 14 in which most patients underwent the procedure 5 to 10 years after diagnosis. Another important finding was a 2.2-year delay between surgical indication and the actual procedure. According to the medical literature, 19 20 functional neurosurgeries such as DBS are generally performed within shorter timeframes than those observed in the current study. The persistent delay observed in our setting is likely multifactorial. First, as the only institution providing DBS within the state public healthcare system, our center receives referrals from across the region. Demand consistently exceeds our surgical capacity, resulting in a persistently-prolonged waiting period for surgery. Given that the annual number of procedures is constrained by governmental funding agreements, the reported waiting time commonly exceeds 2 years. Additional contributing factors include late referral, limited preoperative evaluation capacity, and systemic delays in scheduling surgery. Such delays may be detrimental, as some patients risk losing their surgical window during this time. Unfortunately, we were unable to identify data in the available literature regarding waiting times in other Latin American centers, which prevented direct comparisons.

In our practice, target selection was individualized based on clinical profile, age, and comorbidities, but the STN was preferred in most cases, reflecting institutional experience and expertise. This preference is also supported by evidence 21 showing that STN DBS was associated with greater improvement in off-medication motor symptoms and off-phase functioning compared with GPi DBS, and it enabled larger reductions in dopaminergic medication, while composite scores for cognition, mood, and behavior did not differ significantly between targets. In the present study, we found that motor fluctuations were the most frequent indication, compared with a smaller study 22 in which tremor was the primary indication and motor fluctuations were the second most common. A statistically significant reduction was observed 1 year after DBS surgery, amounting to approximately 25% of the presurgical LEDD. Although not a direct measure of motor or functional outcomes, this reduction reflects a decrease in medication burden, and it may represent an indirect marker of postoperative management optimization, which is consistent with findings reported in other studies. 13 23 We classified the complications as minor and major complications; the main minor complication was wound infection, which is the most frequently reported in the literature, partly due to the fact that the implanted battery and extension cables increase the risk. In our cohort, 7.92% of the patients experienced this complication, a rate higher than those in other reports, which range between 1 and 3%. 24 25 26 The major complications in the current study included: lead migration in 3 patients, intracranial hemorrhage in 2 patients, and deep lead infection and/or meningitis in 2 patients. These represent a rate of 6.93%, which occurred at similar rates to those reported by other centers in South America, Europe, or North America (between 6– 9%). 6 27 In our cohort, all complications were transient, and even major adverse events were not associated with permanent sequelae. A particular issue we observed was battery depletion, a serious event, especially among patients with non-rechargeable systems, related to limited access to timely replacement devices. This problem may be related to the fact that DBS implantation and battery replacement are not routinely covered by the SUS and are frequently dependent on private agreements with limited resources. Although the local government provides a limited number of replacement batteries through the SUS, this supply remains below actual demand. As a result, delays in performing these procedures may occur, leading to adverse events despite the proactive identification by the medical team of patients at risk of depletion. In some cases, patients were required to initiate legal proceedings to expedite device replacement. The literature includes multiple reports of battery-depletion events, largely from case reports and small case series, describing serious clinical deterioration, including parkinsonism. 28 However, to our knowledge, no real-world cohort study has quantified how frequently this event occurs among patients under active follow-up at a single center, particularly in resource-limited settings, where timely battery replacement may be constrained by cost and limited access.

There was a higher proportion of male patients, which is in line with the expected trend in the Brazilian population. 2 Almost all patients were white, and although the majority of the population in the state is also white, this finding reinforces the evidence of limited access to the SUS for black individuals in Brazil compared with other groups. 29 30 The standard deviation for education included the average schooling level of the Brazilian population, which is 10 years according to 2024 data, 31 which is likely lower compared to reference centers in developed countries, where patients undergoing DBS are generally those with higher schooling levels, a factor associated with better outcomes. 32 33 In this cohort, the frequency of family history was lower than that reported in other studies, 34 35 in whicj it ranges from 15 to 21%. Regarding comorbidities, we found psychiatric disorders (depression and anxiety) to be the most frequent associated conditions. These disorders are highly prevalent in PD and may be exacerbated by the disability caused by the disease.

In interpreting the results, it is essential to acknowledge the inherent limitations that may have influenced the findings and their interpretation. First, the retrospective design of the current study represents an inherent limitation, as data were collected from medical records and may be subject to incomplete information and potential selection bias. In addition, standardized outcome measures such as UPDRS scores and quality-of-life assessments were not consistently available, particularly during the early years of the program, and, in some cases, data were missing, which limited the interpretation of DBS-related outcomes. Second, the use of a minimum postoperative follow-up of 3 months represents a limitation of the current study, as most DBS studies 36 37 typically adopt a 12-month follow-up as the primary follow-up time point; however, its impact is likely limited, given that most patients (89.1%) in our cohort had at least 1 year of follow-up. Furthermore, the lack of sociodemographic information restricted our ability to identify potential external factors contributing to the delay in performing the procedure. Regarding genetic testing, only 36% of the patients in this cohort underwent such evaluation; although this is not a mandatory criterion before surgery, it can provide valuable insights into how a patient may respond to DBS. Some forms of monogenic PD are associated with better surgical outcomes than others; 38 however, given our small sample size, this is a finding we cannot definitively evaluate. Regarding strengths, the present study is among the very few in the current literature that depicts the real-world application of this intervention in Latin American populations, where access remains highly limited. To our knowledge, the current is the first study to document this reality within the public healthcare system of a Brazilian city. We consider these findings crucial to understanding the current situation and identifying the necessary directions for the future development of our healthcare systems. However, while resource constraints are a common denominator across most of Latin America, there is significant intercountry variability regarding population ancestry, healthcare resources, and technological infrastructure. Given that this procedure remains technically-inaccessible in some nations, it is difficult to generalize our findings to the entire region.

The present study can serve as a valuable reference for future research, particularly by including additional outcome data to provide a more comprehensive understanding of how these procedures within the SUS, especially considering that, according to current evidence, 39 this intervention is cost-effective compared with medical therapy. The significant delay observed in the diagnosis and the performance of the procedure following its indication is a crucial factor that warrants immediate attention. Strategies must be developed to facilitate timely patient access to DBS while they can still derive maximal benefit, as the indication and effectiveness diminish with disease progression. An interesting finding was the occurrence of battery-depletion events, which are serious episodes that must be prevented. It is essential to understand all contributing factors involved to implement future corrective measures. Addressing procurement logistics, standardizing replacement workflows, and integrating DBS hardware management into public reimbursement structures may significantly reduce preventable adverse events.
